# Intracardic migration of Kirschner wire from the right sternoclavicular joint: a case report

**DOI:** 10.1186/s12893-021-01292-2

**Published:** 2021-06-16

**Authors:** Peng Wang, Cong Chen, Bo Liu, Xiaokang Wang, Wei Jiang, Xiangquan Chu

**Affiliations:** 1 Department of Spine Surgery, Weihai Municipal Hospital, Shandong University, Weihai, Shandong China; 2 Department of Cardiac Surgery, Weihai Municipal Hospital, Shandong University, Weihai, Shandong China; 3 Department of Medical Imaging, Weihaiwei People’s Hospital, Weihai, Shandong China; 4 Department of Orthopedic Surgery, The First Affiliated Hospital of Shandong First Medical University and Shandong Provincial Qianfoshan Hospital, Jinan, Shandong China

**Keywords:** Intracardic migration, Kirschner wire, Trauma, Case report

## Abstract

**Background:**

Migration of wires and pins within the heart is an uncommon complication. Intracardic migration of Kirschner wire can cause several complications.

**Case presentation:**

A 55-year-old male patient was admitted to the emergency service with dyspnea, stabbing chest pain. The patient’s medical history showed that he had undergone a fixation operation using Kirschner wire and plate for treatment of the right sternoclavicular joint dislocation about 5 months prior. Chest computerized tomography revealed a metallic foreign body locating in the pericardium between the aorta and the right ventricle. There were not any serious complications occurred before operation due to the timely detection of potential risks. Removal of the wire was performed via median sternotomy under general anesthesia without cardiopulmonary bypass. The symptoms of dyspnea and chest pain were relieved after surgery, and the patient recovered without any complications.

**Conclusion:**

The Kirschner wire should be used judiciously in amphiarthrosis in orthopedic surgery for the risk of breakage and migration. The possibility of intracardiac migration of wire should be considered when chest symptoms presenting after surgery with the Kirschner wire. Migrated wires must be removed immediately to prevent serious complications. Regular follow-up and early removal of fixation wires are recommended to prevent migration of wires.

## Background

Sternoclavicular joint displacement frequently occurs after traumatic lesions. Kirschner wire (K-wire) is widely used for the treatment of sternoclavicular joint dislocation. Migration of such wire from the clavicle into the heart is extremely rare, but has been known to cause several complications. We present an unusual case that underwent heart migration of a K-wire from the sternoclavicular joint. We discussed the diagnosis, surgical treatment and post-operative outcome. Since very few reports on intracardic migration of K-wire after orthopedic surgery, the present report would provide important academic value.

## Case presentation

**Fig. 1:**
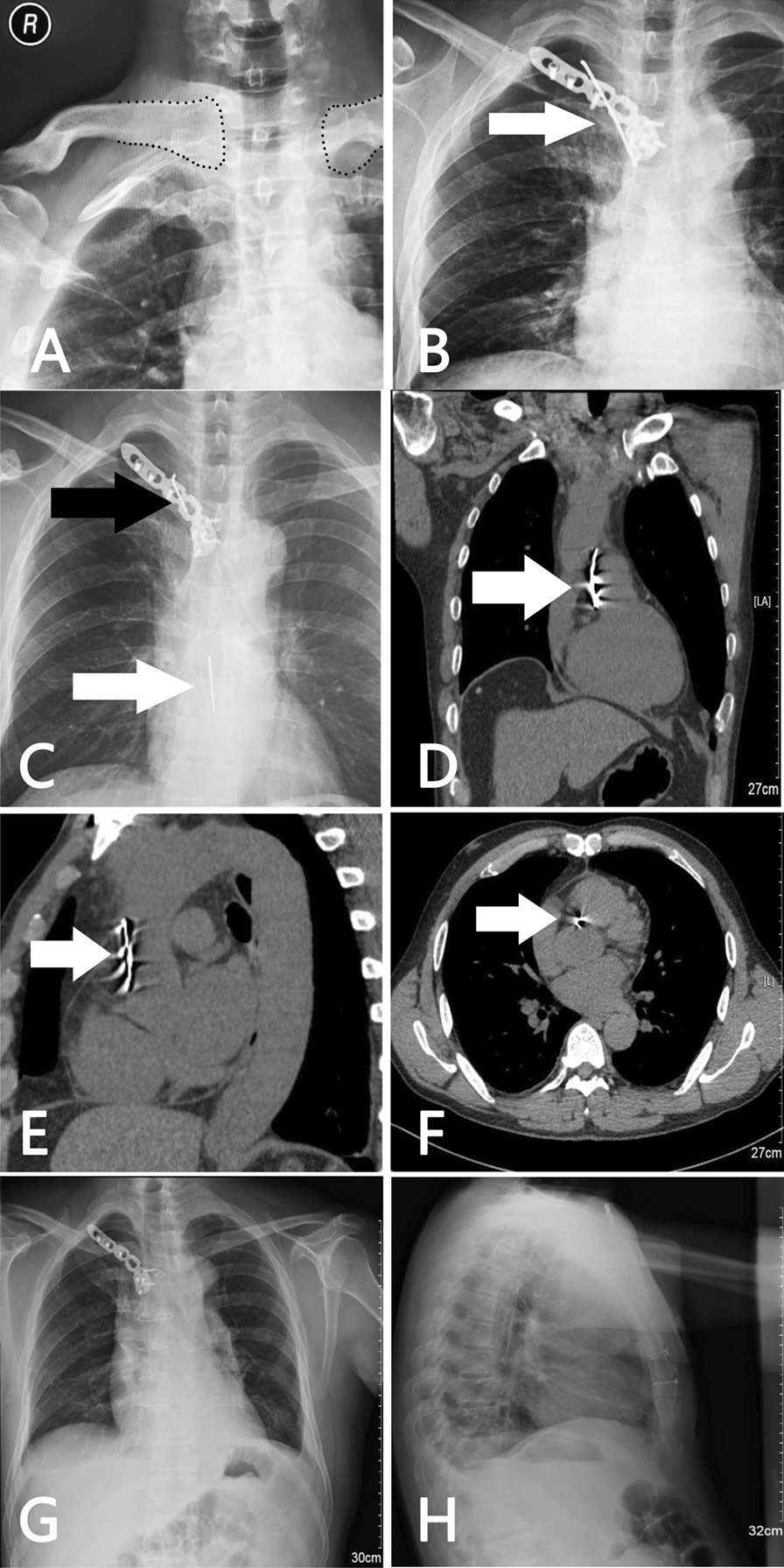
**A** Pre-operative radiograph of the chest showed a dislocation of the right sternoclavicular joint (dotted line). **B** Post-operative radiograph showed reduction of the right sternoclavicular joint with intact K-wire and locking plate. **C** X-ray showing a broken K-wire retained around the sternoclavicular joint (black arrow) and a metallic wire in the cardiac shadow area (white arrow) 5 months after the first operation. Anteroposterior view (**D**) and lateral view (**E**) thoracic computed tomography scan showing a linear metallic wire in the pericardium (white arrow). **F** Coronal view thoracic computed tomography scan showing a migrated foreign body between the aorta and right ventricle (white arrow). Postoperative posteroanterior (**G**) and lateral (**H**) chest radiographs showing the metallic wire was removed

**Fig. 2:**
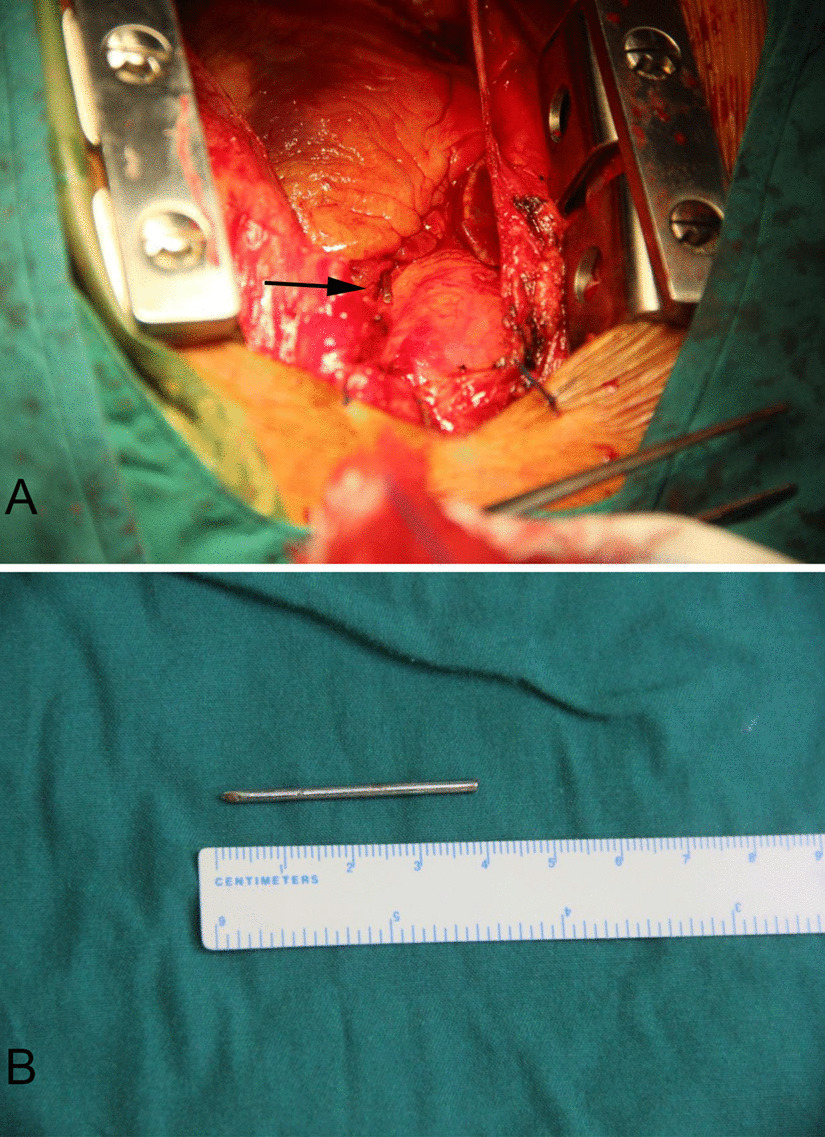
**A** View of the operation field, showing the migrated wire located adjacent to the aorta. **B** The length of the extracted K-wire was 4.0 cm

A 55-year-old male patient was admitted to the emergency service with dyspnea, stabbing chest pain. The symptom had first appeared 3 days earlier. The patient’s medical history showed that he had undergone a fixation operation using K-wire and plate for treatment of the right sternoclavicular joint dislocation about 5 months prior(Fig. 1A, B). His vital signs were stable in physical examination. Chest radiograph showed a broken K-wire around sternoclavicular joint and a linear metallic foreign body in thorax (Fig. 1C). Chest computerized tomography revealed the metallic foreign body in the heart (Fig. 1D–F). There was no evidence of cardiac tamponade or segmental wall motion abnormality in preoperative transthoracic echocardiography. Removal of the wire was recommended and the patient underwent cardiac surgery under general anesthesia via median sternotomy without cardiopulmonary bypass. After pericardiotomy, there was about 50 ml old hematocele in pericardium. The broken K-wire was located between the aorta and the pulmonary artery, behind the right auricle (Fig. 2). Because the wire had a linear shape, it was removed easily. There was an 8 millimeter diameter hematoma caused by K-wire in the anterior wall of the ascending aorta. After clearing the hematoma, the local bleeding tissue was sutured. There was no injury to the heart muscle. The pericardium was closed in standard fashion. There were not any cardiac conduction and rhythm disturbances intra-operatively. Then the K-wire retained around the sternoclavicular joint was removed. The entire intra-operative blood loss was about 100 ml. Follow-up chest radiograph was performed on the third postoperative day (Fig. 1G, H). The patient recovered without any complications and was discharged on the 7th postoperative day. In follow-up 1 year post-surgery, the patient had no particular symptoms such as cough, chest pain and irregular heartbeat.

## Discussion and conclusions

Although many cases of migration of a K-wire for stabilization fracture or dislocation of bones have been described, migration into the heart has been rarely reported. To our knowledge, the first review of metallic pins migration into the heart was documented by Lyons and Rockwood. They referenced four cases of intracardic pin migration as a complication of surgery in the shoulder region [1]. Since then, different cases have been reported in the literature. The sternoclavicular joint fixation using K-wire was the most common site for migration into the heart, followed by the clavicle, the acromioclavicular joint and the proximal humerus [2]. The intracardiac migration of a K-wire from lower limbs has also been reported.

The mechanism of K-wire migration is unclear, but it is probably very slow, allowing the formation of a repairing fibrin layer around the site of lesions. Various theories have been proposed to explain the reason of this migration, including muscle action [3], the freedom of joint movement [4], breathing movement, negative intrathoracic pressure [5], regional bony reabsorption [6] and unthreaded of the pin [7]. In our case, the possible reasons of K-wire intracardic migration may be related to breakage of K-wire caused by micromovement of the thoracoclavicular joint, followed by respiratory and heartbeat movement. In all those reasons, we believe that unbend K-wire or breakage of K-wire is initial cause of K-wire migration. This mechanism reminds that K-wire should be avoided in amphiarthrosis when it is not necessary.

Under any mechanism, the K-wire is able to reach any imaginable site in the body and cause various complications. The most dangerous site where pin migrated is the heart. Once any sign of migration is detected, the surgical procedure may be suggested, because the metallic wire may cause arrhythmia, perforation and sudden death. However, multiple factors should be considered in deciding whether to perform surgical removal, such as the size, shape of the wire and its relationship to the nearby structure as well physical condition of patient [8]. Various methods have been used to remove foreign bodies from the heart, from percutaneous retrieval to open removal, either with or without cardiopulmnonary bypass [9]. In the most cases, removal of intracardiac K-wire requires open heart surgery with cardiopulmnonary bypass, which is helpful to search wire migrated into the myocardial wall [10]. Fortunately, in our case, the wire was long and located in the pericardium, and it was easy for us to remove it without cardiopulmnonary bypass. If the wire was embedded heart cavity, open heart surgery and the subsequent repair using cardiopulmnonary bypass is required [11].

To prevent migration of the K-wire, operators must follow K-wire implant handling protocol. K-wires should be removed upon achieving their goal of temporary fixation intraoperatively. If the K-wires need to retain to fix fracture or dislocation, it is strongly advocated to bend the wire to prevent migration [12]. However, there is still a possibility of loosening or breakage of K-wires in the postoperative period due to osteopenia or joint movement [13]. Rigorous radiography and clinical follow-up every 2–4 weeks must be performed to evaluate bone healing and position of internal fixation. It should be removed immediately as soon as K-wire is found broken, or migrating, or bone healing is achieved 4–6 months later in the follow-up.

In conclusion, our report emphasizes the risks of K-wire using in fixation dislocation and fracture. Surgeons should be realizing this potentially serious complication of K-wire migration. Several suggestions may be followed: (1) The K-wire should be used judiciously in amphiarthrosis in orthopedic surgery; (2) the patients should have regularly clinical and radiographical followed-up after surgery; (3) fixation should be removed on time once the fracture or dislocation is considered stable; (4) K-wire should be removed immediately after migration or breakage is recognized, even if none any symptoms were present.

## Data Availability

The data supporting the conclusions of this article are included within the article and its figures.

## Abbreviations

K-wire: K-wire
cm: Centimeter
ml: Milliliter
